# Persistent High Level of Urinary Tumor Marker Carbohydrate Antigen 19-9 in Prenatally Diagnosed Dysplastic Kidney

**DOI:** 10.1155/2014/259870

**Published:** 2014-12-31

**Authors:** Reza Khorramirouz, Maryam Ebadi, Fatemeh Rahimi Sherbaf, Abdol-Mohammad Kajbafzadeh

**Affiliations:** ^1^Pediatric Urology Research Center, Children's Hospital Medical Center, Pediatric Center of Excellence, Tehran University of Medical Science, No. 62, Dr. Gharib's Street, Keshavarz Boulevard, Tehran 1419733151, Iran; ^2^Department of Obstetrics and Gynecology, Mirza Koochackkhan Women's Sciences Hospital, Tehran University of Medical Science, Tehran 1419433151, Iran

## Abstract

Tumor marker carbohydrate antigen 19-9 (CA 19-9) level has gained clinical significance in gastrointestinal malignancies and in various solid and cystic diseases. Dysplastic kidney is a congenital abnormality resulting from atresia of the ureteral bud during the embryogenesis which can be unilateral or bilateral. We report unilateral dysplastic kidney with extremely large cyst diagnosed by routine ultrasonography in the 32nd week of gestational age with high levels of CA 19-9 in cystic and amniotic fluid, as well as persistent high urinary levels of this tumor marker during the 1-year follow-up. Persistent high urinary CA 19-9 level even after cyst aspiration may be attributable to remained function of dysplastic kidney due to remained epithelial lining.

## 1. Introduction

Tumor marker CA 19-9 level has gained clinical significance in evaluating gastrointestinal malignancies especially pancreatic pathologies. In addition, its diagnostic and prognostic values in other cases have been evaluated. Increased cystic fluid or serum CA 19-9 levels have been described in various cystic fluids including hepatic, splenic, esophageal, and parathyroid cysts [1–4].

The diagnostic value of CA 19-9 level in genitourinary tract abnormalities has been reported in urothelial carcinoma [5]. Our previous report has elucidated the association of increased urinary and serum CA 19-9 levels and congenital ureteropelvic junction obstruction (UPJO) in children [6], indicating the significance of this tumor marker in such benign genitourinary conditions.

Multicystic dysplastic kidney (MCDK) is a common congenital abnormality originating from incomplete ureteral bud development during embryogenesis. This congenital anomaly (MCDK) has the incidence of approximately 1 : 4300 live births affecting males more than females [7–9]. Most cases of MCDK are detected during fetal ultrasound by the 15th week of gestation. It can be unilateral or bilateral and is classified as simple or complex.

Evaluation of the cystic content and also a urinary marker in MCDK has not been well described in the literature and no study has previously focused on urinary levels of CA 19-9 in dysplastic kidney. This is the first case report of tumor marker CA 19-9 measurement in fetal cystic fluid and immediate postnatal urine of an infant with MCDK. Herein, we report the first case of unilateral MCDK containing an extremely large cyst with elevated urinary and cystic fluid levels of tumor marker CA 19-9 and persistent high urinary CA 19-9 levels even after cyst aspiration until 1 year of follow-up.

## 2. Case Report

**Figure 1:**
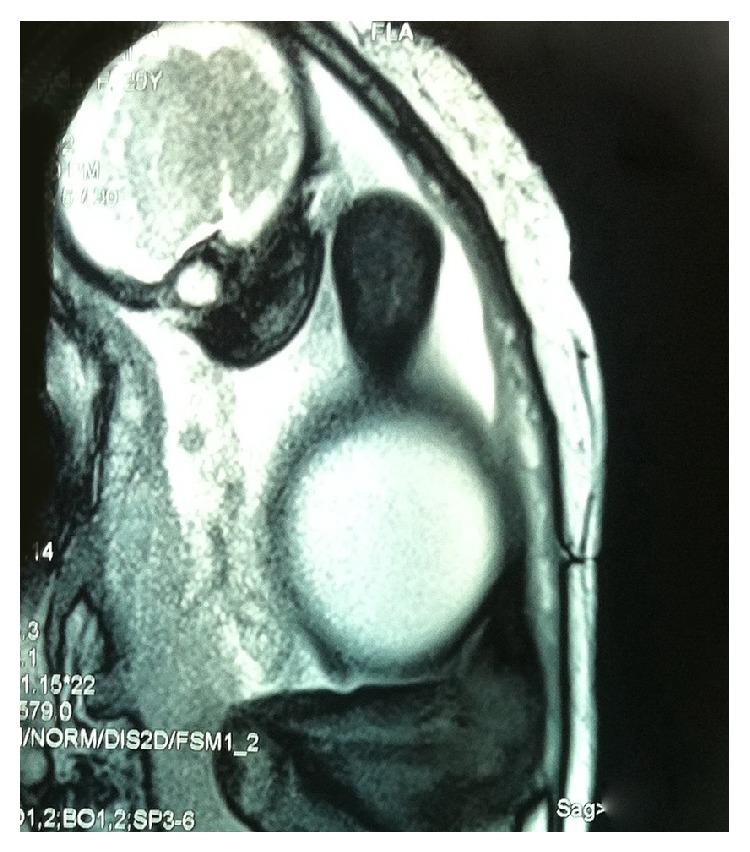
Magnetic resonance urography was done in 34th week of gestational age which shows a large cystic mass measuring 8∗9 cm in the right abdominal part above the right kidney.

**Figure 2:**
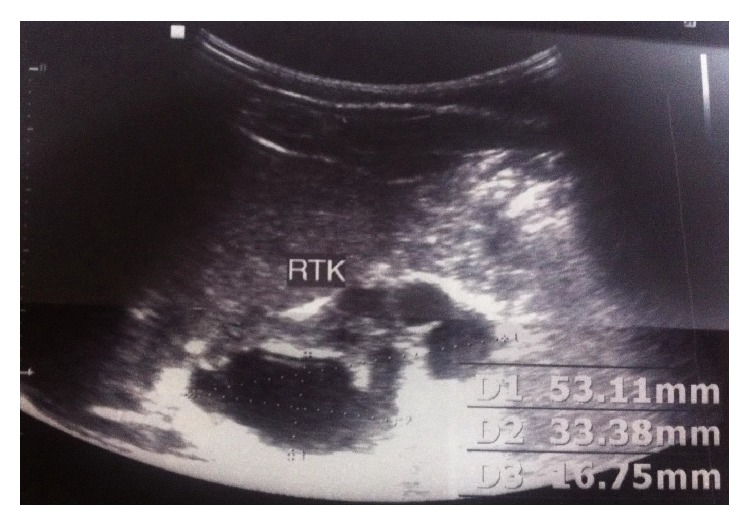
Sonography was done in 6th day which shows a right dysplastic kidney with the largest cyst size measuring 33∗16 mm after cyst aspiration.

**Figure 3:**
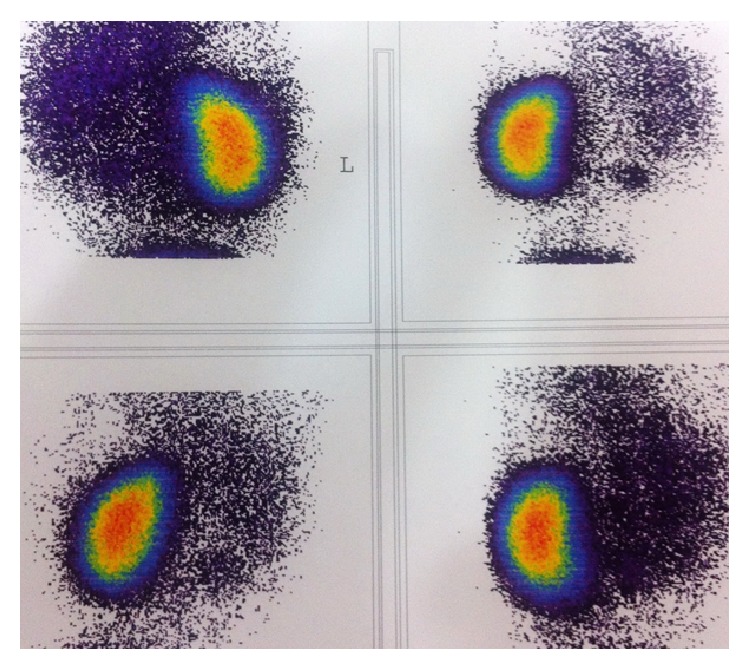
DMSA scan showed severely decreased right kidney uptake that is compatible with right dysplastic kidney with normal left kidney function.

A thirty-year-old pregnant woman was referred to the pediatric urology center following a suspicious finding on routine screening sonography performed in 32 weeks of gestational age. Ultrasonography evaluation revealed a large cystic mass in the right abdominal cavity with right dysplastic kidney without oligohydramnios in the fetus. Fetal magnetic resonance urography (MRU) which was performed in the 34th week of gestation confirmed a large cystic fetal abdominal mass (8∗9 cm) in the right abdominal cavity which displaced bowel loops to the left (Figures 1 and 2). The mass was suspicious for a large single mesenteric cyst. Prenatally, the patient underwent cystic fluid aspiration (400 mL) which resulted in cyst shrinkage; the sample was evaluated for the tumor marker CA 19-9 and revealed highly increased levels. The fresh fetal cyst fluid was transferred to our human stem cells laboratory for fetal cell separation and culture in order to characterize the cells origin which was nonconclusive. With the cyst collapse after aspiration and further imaging study, diagnosis of probable mesenteric cyst was excluded and the MCDK with normal contralateral kidney was depicted. Six days after birth the patient was evaluated by urinary tract ultrasonography in which a small right kidney with increased parenchymal echogenicity and multiple cysts was reported suggestive of right dysplastic kidney with a normal left kidney. In order to rule out possible concomitant genitourinary abnormalities, the patient underwent direct radionuclide cystography by injection of 99 m Tc-pertechnetate into the bladder according to the estimated bladder capacity for weight, which showed no vesicoureteral reflux in both sides. To determine the renal function, dimercaptosuccinic acid (DMSA) scan was performed which illustrated severe diffusely decreased tracer uptake in the right kidney with acceptable left kidney uptake (6.1% and 93.9% function in right and left kidneys, resp.) (Figure 3). The important finding in this case was a high cystic fluid CA 19-9 level (more than 1000 U/mL) before birth. At the time of delivery samples of amniotic fluid and urine were analyzed which had levels of CA 19-9 greater than 1000 U/mL in both samples. The patient underwent various follow-up visits by urinary CA 19-9 level 50 and 70 days after birth, which had level of 832 and more than 1000, respectively. During the first year, the patient had persistent increased values of CA 19-9 with dysplastic right kidney as evaluated by ultrasonography. Overall the patient had increased levels of CA 19-9 in cystic fluid, amniotic, and urine samples right after birth with refractory high urinary levels in a one-year follow-up visit despite the cyst shrinkage.

## 3. Discussion

This case represents the first patient with prenatally diagnosed MCDK with a prominent large cyst containing high levels of CA 19-9 which remained elevated in the one-year follow-up visits after birth despite cyst shrinkage.

Most studies have focused on the level of this tumor marker in malignant cases mostly gastrointestinal tumors; but in rare cases urological condition such as bladder and renal pelvic carcinoma have been associated with increased CA 19-9 levels [10, 11]. The prognostic value of this tumor marker was also reported in some benign urological conditions including hydronephrosis and UPJO [6, 12]. Remarkably, the present case represents a different pattern compared to previous studies; it is the first report of MCDK with high levels of CA 19-9 level in cystic fluid, postpartum first voided urine, and amniotic fluid. The increased level of CA 19-9 in other cysts including spleen, esophageal, hepatic, and parathyroid cysts is not comparable to the prominently high levels observed in this case (more than 1000 U/mL) [1–4, 13]. On the other hand, the high levels of CA 19-9 either in cystic content or serum returned to normal levels after surgical resection during several weeks; conversely in this case persistent high levels of urinary CA 19-9 were observed despite surgical aspiration.

Previous studies by our team have elucidated the role of epithelial cells in elevated CA 19-9 levels [6]. Moreover, they have previously reported increased CA 19-9 levels in the amniotic fluid and first urine of patients with posterior urethral valves [14] as well as the serum and urine of patients with UPJO [6]. Therefore, to rule out other probable congenital urological abnormalities as the cause of persistent high levels of CA 19-9, proper diagnostic evaluations were performed, all of which were negative. The authors hypothesize that persistent high urinary levels of this tumor marker in this case may be attributed to both renal cyst and the existence of some function parenchyma in between the cysts of right dysplastic kidney. This hypothesis theoretically might be confirmed by presence of 6.5% functioning tissue in the MCDK. Although the cystic content had high levels of CA 19-9 leading to high levels in amniotic fluid and urine, persistent elevated urinary level of this tumor marker after birth and cyst shrinkage could be explained by the presence of some concomitant renal parenchymal tissue in right dysplastic kidney. Based on findings from DMSA scan, the right dysplastic kidney had 6.1% activity; hence, it can be proposed that the epithelial lining as well as the decreased activity in the dysplastic kidney could have resulted in persistence in CA 19-9 level in the patient after 1 year of follow-up. On the other hand, previous studies in the literature reporting increased CA 19-9 levels in other cysts have revealed return to normal range after cyst removal, possibly due to complete resection of the cystic content and epithelial lining in such cases [2, 3]. This explanation is compatible with highly persistent level of CA 19-9 in this patient, since the cyst was only aspirated and epithelial lining and scanty functioning renal tissue remained intact, possibly resulting in persistent elevated level of this tumor marker in urine.

## 4. Conclusion

To the best of our knowledge, this study is the first reported case of MCDK with persistent high levels of CA 19-9 in cystic content and voided urine. This finding may pave the road to future studies on the diagnostic and prognostic value of CA 19-9 in MCDK and other congenital renal anomalies.
